# Cks1: Structure, Emerging Roles and Implications in Multiple Cancers

**DOI:** 10.4236/jct.2013.48159

**Published:** 2013-10-29

**Authors:** Vinayak Khattar, Jaideep V. Thottassery

**Affiliations:** 1 Southern Research Institute, Birmingham, USA; 2 University of Alabama Comprehensive Cancer Center, Birmingham, USA

**Keywords:** Cks1, Cks2, Skp2, Cdk1, p27, Kip1, p130, Rb2, CKI, Ubiquitination, Proteasome, ERK1/2

## Abstract

Deregulation of the cell cycle results in loss of normal control mechanisms that prevent aberrant cell proliferation and cancer progression. Regulation of the cell cycle is a highly complex process with many layers of control. One of these mechanisms involves timely degradation of CDK inhibitors (CKIs) like p27^Kip1^ by the ubiquitin proteasomal system (UPS). Cks1 is a 9 kDa protein which is frequently overexpressed in different tumor subtypes, and has pleiotropic roles in cell cycle progression, many of which remain to be fully characterized. One well characterized molecular role of Cks1 is that of an essential adaptor that regulates p27^Kip1^ abundance by facilitating its interaction with the SCF-Skp2 E3 ligase which appends ubiquitin to p27^Kip1^ and targets it for degradation through the UPS. In addition, emerging research has uncovered p27^Kip1^-independent roles of Cks1 which have provided crucial insights into how it may be involved in cancer progression. We review here the structural features of Cks1 and their functional implications, and also some recently identified Cks1 roles and their involvement in breast and other cancers.

## 1. Introduction

Regulation of the cell cycle is crucial for cellular proliferation and survival [1,2]. The engines that drive the cell cycle include cyclin dependent kinases (CDKs) and their activating cyclin subunits which oscillate during the cell cycle and thus modulate the activity of CDKs at specific points in various cell cycle phases [3]. Another level of control requires the action of cyclin dependent kinase inhibitors (CKIs) on CDKs [4]. These CKIs, along with other cell cycle substrates are regulated by precise and coordinated proteasomal degradation [5,6]. This in turn can regulate the activities and abundance of other substrates [5,6].

The ubiquitin proteasomal system (UPS) employs a series of activating (E1), conjugating (E2) and ligase (E3) enzymes and appends ubiquitin chains to substrates, and directs them to timely degradation by the proteasomal machinery [7]. For instance a crucial step in regulating mammalian G1-S transition is one which involves the degradation of p27^Kip1^ by the UPS, which is required for full activation of cyclin E-CDK2 complexes [8,9]. Following its phosphorylation on Thr-187 by cyclin E-Cdk2, p27^Kip1^ is ubiquitinated by the SCF-Skp2 E3 ligase [10]. Ganoth *et al.* demonstrated that fully reconstituted SCF-Skp2 only ubiquitinates p27^Kip1^ when it is supplemented with Cks1 [11,12]. Investigations revealed that Cks1 interacts with the substrate recognition component in this complex, Skp2, and facilitates its p27^Kip1^ ubiquitination activity [12,13]. Until recently this was the only well characterized molecular role for Cks1 in mammalian systems. However, emerging research reveals many more diverse and p27^Kip1^ independent roles of Cks1 that encompass growth signaling pathways [14–25], apoptosis [25] and even DNA damage responses [26,27].

Cks1 was discovered in 1986 in a screen that identified genes that allow temperature sensitive cdc2 mutants in yeast to grow at the restrictive temperature [28–30]. The screen identified a molecule called Suc1 (Suppressor of cdc2) which when present on multicopy plasmids in *Schizosaccharomyces pombe* could rescue cells mutated in their cdc2 [28]. The study hinted that the action of-Suc1 was specific to cdc2 suggesting direct interaction between the two [28,29]. Indeed it is now recognized that Cks1 (Suc1 in *S. pombe*) does interact with CDKs at a site that is distinct from their ATP binding or cyclin binding sites [31–33]. Further characterization revealed that overexpression or mutations in Suc1 caused defects in the cell cycle and in viability showing that this gene was crucial for cell cycle progression [34].

## 2. Structure and Functional Implications

### 2.1. Sequence, Secondary and Higher Order Structure

The sequence of the Cks family of proteins is highly conserved across species [31,35,36]. Exploiting this similarity Richardson *et al.* designed degenerate primers allowing them to clone the human orthologs CksHs1 and CksHs2 from a HeLa cDNA library [37]. Comparison of CksHs1 and CksHs2 reveals 81% identity between the two molecules [37]. The evolutionary logic behind this conservation can be appreciated in the context of the crucial role of Cks1 in cell cycle and its interactions with CDKs [38]. For instance both human Cks proteins have identities higher than 50% when compared with both the fission and budding yeast Cks sequences and are capable of rescuing a null mutation in the *S. cerevisiae* Cks1 gene [33,37].

Although there is a high level of conservation among Cks1 sequences across species, the length of Cks1 in *S. Cerevisiae* and *S. pombe* is 150 and 113 residues respectively, while the human orthologs, CksHs1 and CksHs2, are both 79 amino acids long [31,35,36]. The major differences that account for this difference in length are two extensions at the N and C-terminals and a 9 amino acid insertion sequences in yeast Cks sequences not found in CksHs1 or CksHs2 [32]. The C-terminal extension in yeast sequences includes a 16 residue long polyglutamine tail and it has been observed that these Cks1 molecules can form fibrillar aggregates characterized by presence of specific hydrogen bonding between polyglutamine sequences [35,36,39]. The characteristics of these aggregates were found to be very similar to those observed in amyloid fibrils or aggregates observed in other polyglutamine deposition diseases on firm that you have the correct template for your paper size.

CksHs1 and CksHs2 have key structural differences at higher levels of structural organization despite their remarkable sequence identity. Arvai *et al.* determined the structure of Cks1Hs1 at a resolution of 2.9 A, and observed that the domain architecture and subunit conformation for CksHs1 and Cks1Hs2 are dramatically different [31]. Although theoretically both CksHs1 and CksHs2 can exist in monomeric and dimeric forms *in vitro*, it appears that Cks1Hs1 is more stable in monomeric form whereas CksHs2 was observed in primarily dimeric and even hexameric forms [31]. *In vivo* it is predicted that binding of CDKs and metal ions influences that stability and predominance of a particular form over the other [31,40].

Nevertheless CksHs1 does crystallize as a dimer with anionic cofactors like vanadate, tungstate or phosphate [31,40]. The CksHs1 dimer utilizes a crucial hydrogen bond between residues Tyr 8 present in the *β*1 strand of each molecule [31]. The Cks1Hs1 monomer is a single polypeptide chain folded in a single domain comprising four anti-parallel *β* sheets sandwiching two *α*-helices [31]. Starting from the N-terminus the sequence of secondary structural elements includes two anti-parallel *β* sheets *β*1 and *β*2 followed by two short *α* helices and finally two anti-parallel *β* sheets *β*3 and *β*4 [31]. Following crystallization these monomers can assemble into dimeric form with eight *β* sheets forming a twisted structure due to a tilt of 50 degrees between the two adjacent *β* strands [31]. Despite the fact that both CksHs1 and CksHs2 molecules can form dimers, the folding of a small sequence conserved region between Glu 61 and His 65 results in dramatically different conformations for the *β*4 strand resulting in he characteristic *β* strand exchange form observed in CksHs2 [31]. Thus, depending on the conformation of this highly conserved region forming a *β*-hinge between *β*3 and *β*4 strands, Cks proteins can exist in two forms [31]. When this hinge region is in an extended conformation, it facilitates the *β*4 strand from one monomer to fold out and interact with secondary structural elements of the other monomer [31]. Similarly the corresponding *β*4 strand from the second monomeric domain extends out and “squeezes” itself between secondary structural elements on the other monomer of the Cks molecule thus resulting in characteristic structure termed *β* strand exchange dimer [31]. On the other hand in a closed conformation this hinge region is more ordered and will exist as a *β* bend preventing any such strand exchange between monomers [31]. In CksHs1 the *β* hinge region is in this closed conformation and hence does not allow any *β* strand exchange between the monomers [31]. On the other hand this conserved region is conformationally different in CksHs2 allowing it to assemble as a *β* strand exchanged dimer [31].

These subtle differences in the *β* hinge region that leads to dramatically different subunit conformation suggests a theory where a specific stimulus or cell cycle event may trigger a change from one form to another as a part of a regulatory mechanism [31]. This in turn could lead to changes in critical interactions between Cks and its partner proteins leading to other downstream physiological consequences [31]. Although, whether in fact such changes in domain architecture of CksHs1 do play a part in cell physiology remains speculation for the time being, Arvai *et al.* have pointed out that critical residues such as Tyr 12, Tyr 19 and Tyr 57 are exposed in the single domain fold whereas these regions are masked in dimeric or hexameric forms [31].

### 2.2. Functional Implications of Structural Features in Cks1

Functionally speaking Cks1 structure is characterized by the presence of three regions including a cdc2/CDK binding region, a Skp2 binding region and an anion/phosphate binding pocket [31,32,41–43] (see Table 1 for the key residues mediating the interactions in these functional regions). Another striking feature of Cks1 structure is that the CksHs1 molecule shows a strong homology to the N-lobe domain of CDK2 [31,32,41–43]. That Cks1 binds to cdc28 and cdc2 was already established during early coimmunoprecipitation studies with yeast homologs of Cks1 [44]. Similarly it was later demonstrated that Xe-p9 could also interact with Cdc2/cyclinB complexes [45]. The crystal structure of human CDK2 in complex with CksHS1 revealed that Cks1 utilizes all four beta sheets for this interaction and binds specifically to the C-terminal lobe of CDK [32]. Also it was revealed that the CDK domain does not interact with the cyclin binding sites or regulatory phosphate binding sites [32]. Cks1 can bind to CDK only in its monomer conformation and uses specific hydrophobic residues (see Table 1) as well as the conserved *β*-hinge region [32]. On the other hand the opening of this *β* hinge, which is the characteristic feature of the beta strand exchanged dimer does not allow Cks to bind to CDK2 [32].

The ternary complex of Skp1-Skp2-Cks1 in association with a phosphorylated p27^Kip^ peptide elucidated by Pavletich *et al.* has provided significant insights into how Cks1 influences recognition of p27^Kip1^ by the SCF complex [42]. It is a sickle shaped binary structure where Skp1 and the F-box domain of Skp2 form the handle and the Skp2 leucine rich repeat (LRR) domain forms the curved blade [42]. The C-terminal tail of Skp2 at the end of tenth LRR curves inwards and interacts with first LRR. The Cks1 molecule docks into this concave groove formed by the LRR and C-terminal tail of Skp2. Three key residues (see Table 1) of Cks1 are required for H-bonding interactions with Skp2 and map into the 2 helix of Cks1 [41,42]. Phosphorylated p27^Kip1^ is recognized by phosphate binding pocket of Cks1 and p27^Kip1^ forms regions of contacts with both Cks1 and Skp2 by inserting a crucial Glu185 residue in the interface formed between Cks1 and Skp2 [41,42]. Hydrogen bonding between highly conserved Skp2 residues (Trp265, Arg294, Asp319, and Arg344 side chains) and those of Cks1 (Ser41, Glu40, and Asn45 side chains and Ser41 carbonyl group) is a crucial prerequisite for Cks1-Skp2 interaction and hence altering these Cks1 residues is known to compromise the p27^Kip1^ ubiquitination activity of the SCF complex [41,42]. The residues Ser41 and Asn 45, are replaced by Glu and Arg residues in CksHs2 precluding its interaction with Skp2 [41,42]. Yeast Skp2 is not known to interact with Cks1, although Cks1 in yeast does play a role in the multi-site phosphorylation of Sic1, the CDK inhibitor that is homologous to mammalian p27^Kip1^ [46].

The ubiquitination of p27^Kip1^ is preceded by its phosphorylation at residue Thr^187^ which is then recognized by the phosphate binding pocket present in Cks1 [31,41,42, 47,48]. Two anion binding pockets are present near the dimer interface within a crevice formed by the dimer association [31]. In fact negatively charged moieties like vanadate or tungstate were used to facilitate crystallization of CksHs1 by Arvai *et al.* [40]. Crucial van der Walls contacts and hydrogen bonding contacts from residues Lys11, Arg20 and Arg71 and to the main chain nitrogen atom of Ser51 stabilize the interaction with these moieties [42].

## 3. Diverse Cellular Functions of Cks1

Cks1 roles can be broadly classified into four categories: 1) roles in modulating cell cycle, 2) roles in transcription, 3) roles in growth signaling pathways, and 4) other emerging roles. Excellent reviews describing some of these functions of Cks1 are available [49–53].

### 3.1. Cks1 Roles in Modulating the Cell Cycle

The evidence for Cks1 modulating the cell cycle was provided by Tang and Reed where it was reported that loss of Cks1 in *S. cerevisiae* causes defects in both G1-S and G2-M transition [54]. However the mechanism by which Cks1 regulates G1-S transitions in mammalian systems was elucidated in two independent reports by Ganoth *et al.* and Spruck *et al.* which showed that Cks1 is indispensable for proteasomal degradation of p27^Kip1^ [12,13]. Ganoth *et al.* showed that a fully reconstituted SCF-Skp2 complex cannot ubiquitinate p27 *in-vitro* unless it was supplemented by a crucial factor (Factor I) derived from the unbound fraction of the HeLa cell extracts fractionated on DEAE–cellulose columns [12]. Further purification and characterization of this unknown factor revealed that this factor was Cks1 [12]. Furthermore this report established that Cks1’s role in p27^Kip1^ degradation was specific to the SCF-Skp2 ligase since it had no impact on the activity of other ubiquitin ligases like SCF *β*-TrCP [12]. Spruck *et al.* utilized MEFs derived from Cks1^−/−^ mice [13]. Cks1^−/−^ mice exhibit a smaller body size, which is possibly due to proliferative abnormalities (due to accumulation of p27^Kip1^ in somatic cells during embryonic development) [13]. Interestingly the roles of Cks1 in p27^Kip1^ degradation have been suggested to be independent of its interactions with CDKs. A Cks1 mutant defective in CDK binding (E63Q) has been shown to be capable of p27^Kip1^ ubiquitination [13]. On the other hand other mutations in the CDK2 binding site of Cks1 do reduce the ability of cyclin A-CDK2 to promote p27^Kip1^ ubiquitination [41]. Although phosphorylation of p27^Kip1^ on residues Thr-187 by cyclin E/A-CDK2 complex is a crucial prerequisite for its recognition and proteasomal targeting by the SCF-Skp2 ubiquitin ligase, whether the interactions of Cks1 with CDK2 itself have any impact on its p27^Kip1^ ubiquitination activity remains controversial [13,41,42].

Three models have been suggested to describe the role of Cks1 in p27^Kip1^ ubiquitination [50]. The first model suggests that binding of Cks1 to the C-terminal of Skp2 results in a necessary conformational change that allows the Skp2 to interact efficiently with its phosphorylated substrate, p27^Kip1^. This model depicts a binding site of Cks1 wherein Cks1 docks in a concave groove formed by the LRR region and the C-terminal tail of Skp2 [42,50]. It is suggested that interaction of Cks1 with the C-terminal tail region of the Skp2 molecule “nudges” the occluding LRR region and allows efficient interaction between LRR and the phosphorylated substrate [42,50,55]. The second model suggests that Cks1 may physically act as an adaptor or bridge between phosphorylated substrate p27^Kip1^ and F-box protein Skp2 [42]. It is possible that the phosphate binding site of Cks1 recognizes phosphorylated p27^Kip1^ which is then brought in close proximity to Skp2 for ubiquitination [42]. Although it has been shown that Cks1 interaction with CDK2 is not necessary for p27^Kip1^ degradation, it has been suggested in a third model that Cks1-CDK2 binding pulls out the p27^Kip1^-cyclin A-Cdk2 complex and favors its interaction with SCF-Skp2 complex [42]. Although further genetic and biochemical studies are required to distinguish between these possibilities, the role of Cks1 in p27^Kip1^ degradation is indisputable.

Cks1 also regulates the proteasomal degradation of p130/Rb2 which is both a pocket protein and an inhibitor of CDK2 [56,57]. The expression of p130 is largely restricted to G0 phase of the cell cycle [56,57]. Like p27^Kip1^ the turnover of p130 is regulated proteasomally [58]. As proliferating cells enter from G0 phase to G1, p130 gets phosphorylated by CDK4/6 complexes and is proteasomally degraded by a process which employs the SCF-Skp2 E3 ubiquitin ligase complex [58]. In fact loss of either Skp2 or Cks1 impairs proteasomal stability of p130 as evidenced by its accumulation in asynchronous or thymidine arrested Skp2^−/−^ and Cks1^−/−^ fibroblasts [58]. Thus Cks1 is responsible not only for regulating the p27^Kip1^ degradation but also plays a pivotal role in determining p130 stability in proliferating cells [58].

Cks1 has also been implicated in the degradation of mitotic substrates by regulating the APC/C ubiquitin ligase and spindle assembly checkpoint (SAC), which is crucial for orchestrating mitotic timing [59–62]. The SAC fine-tunes the timing of proteasomal degradation through APC/C ubiquitin ligase by inactivating its coactivator Cdc20 [63,64]. This in turn prevents premature degradation of APC/C mitotic substrates like securin and cyclin B1 ensuring accurate sister chromatid separation before mitotic exit [63,64]. However other APC/C substrates like cyclin A are degraded even though the SAC is still active [65]. Studies by Di Fiore *et al.* and Wolthius *et al.* have explained this paradox by showing that the N-terminus of cyclin A competes with SAC proteins to bind to Cdc20 [66,67]. Following this Cks1 recruits the cyclin A-cdc20 complex to a phosphorylated APC/C ubiquitin ligase, triggering cyclin A ubiquitination and subsequent proteasomal degradation. [66,67]. Furthermore it was demonstrated that the anion binding site of Cks1 is crucial for the SAC independent degradation of cyclin A [66,67].

Loss of Cks1 also results in impaired mitotic passage in yeast and in *Xenopus* egg extracts [45,54]. In *Xenopus* egg extracts, Cks1 is crucial for dephosphorylation of Y15 residue of CDK1 [45]. Cks1 promotes MPF dependent phosphorylation of cdc25c phosphatases, wee 1 and myt1 kinases, all molecules that participate in CDK1 activation by removing inhibitory phosphorylation from Cdk1 [45]. Xe-p9 (*Xenopus* homologue of Cks1) is also known to promote cyclin B1 degradation [68,69]. More specifically Xe-p9 regulates mitotic exit by stimulating phosphorylation of cdc27, another important component of the APC/C complex that targets cyclin B1 and several other mitotic molecules for proteasomal degradation, thus orchestrating mitotic exit in these cells [68,69]. Studies in our laboratory have shown that loss of Cks1 in MCF-7 breast cancer cells leads to blockade in mitotic entry with corresponding loss of CDK1 [70]. Furthermore the mitotic block can be rescued by reintroduction of exogenous CDK1 [70]. A report by Martinsson-Ahlzen *et al.* has also demonstrated that loss of Cks1 in MEFs and HeLa cells results in cell cycle arrest in G2 phases which can be reversed reintroduction of cyclin B1 [71]. Interestingly Cks1 loss does not alter cell cycle profiles in human mammary epithelial cells (HMECs) or normal human lung fibroblasts [70,72].

Although G1-S defects following Cks1 loss in mammalian cells can be accounted for by p27^Kip1^ accumulation, recent reports have indicated that some defects can also be explained in part by p27^Kip1^ independent mechanisms [73]. In studies reported by Keller *et al.* it was found that CDK1 kinase activity of the Cks1^−/−^ MEF cells was not altered whereas there is considerable loss in CDK2 kinase activity [73]. Furthermore concomitant loss of p27^Kip1^ in Cks1^−/−^ MEF did not rescue this loss in Cdk2 kinase activity indicating that part of G1-S defects observed are p27^Kip1^ independent and a direct conesquence of Cks1 interaction with CDK2 activity [73]. In fact a report by Liberal *et al.* has shown that Cks1 can override DNA damage response by increasing CDK2 kinase activity and overriding the inhibitory effects of Y15 phosphorylation on CDK2 [27].

### 3.2. Cks1 Roles in Transcription

Recently Cks1 has also been implicated in transcriptional regulation [71,74–77]. In fact some of these transcriptional roles also indicate other ways by which Cks1 might be involved in regulating cell cycle transitions [71, 76]. For instance, Cdc20 has been shown to be a transcriptional target of Cks1 [76]. Cdc20 is a regulator which associates with and modulates the activity of the APC ubiquitin ligase during distinct phases of cell cycle [78]. It was shown that Cks1 is essential for dissociation of Cdc28 from Cdc20 promoter and recruitment of specific proteasomal subunits like Rpt1, *Pre*1 on this promoter [76]. It has been suggested that this periodic association and dissociation events on the Cdc20 promoter facilitates remodeling of transcriptional complexes docked on the Cdc20 promoter [76]. It has also been recently reported that that Cks1 plays a role in GAL1 transcription, whereby Cks1, CDK1 and the 19S subunit of the proteasome are recruited to the GAL1 promoter, specifically attaching to the histone H4 amino-terminal tail of the chromatin [74]. This activity has been reported to alter nucleosome density and evict nucleosomes from the chromatin region thereby inducing GAL1 transcription [74]. Cks1 has also been shown to transcriptionally regulate the expression of *cdc*2, *cyclin B* and *cyclin A* in mammalian cells [71].

The regulation of Cks1 gene expression itself is now known to be regulated through transcriptional mechanisms [79–87]. Cks1 mRNA levels start rising in late G1 reaching a peak before the onset of S-phase [88]. Another peak is observed at G2/M phase of the cell cycle [37]. Various reports have provided insights into possible transcriptional regulators for Cks1 [79–86]. Mutating a potential CDE/CHR (cell cycle dependent element and cell cycle genes homology region) tandem repeat within Cks1 promoter compromises transcriptional activation of Cks1 indicating that this element acts as a possible transcriptional regulator [87]. Although Cks1 does not have p53 binding site, forced induction of p53 represses mRNA and protein expression of Cks1 possibly due to p53 dictated repression of Cks1 promoter [87]. On the other hand NF-Y, FoxM1, and Myc are known to act as transcriptional activators for Cks1 [80,81,83,85,87]. In fact Myc induced Cks1 activation has been proposed to be a switch that triggers p27^Kip1^ loss and subsequent cell proliferation and tumorigenesis by Keller *et al.* [81].

In primary T-lymphocytes CD28 along with T-cell receptor (TCR) provides a co-stimulatory signal that is required for T cell activation and entry into S-phase [89, 90]. CD28 co-stimulatory signals downregulate p27^Kip1^ by inducing transcription of Skp2 and Cks1, thus enhancing its proteasomal degradation [79]. TGF-treatment of mink lung epithelial cells and Hep3B cells also decreases Cks1 mRNA transcripts, and this loss of Cks1 has been known to compromise p27^Kip1^ ubiquitination, and also triggers Skp2 autoubiquitination and degradation [86]. Furthermore, B-Raf and cyclin D1 also downregulate Cks1 expression at the mRNA and protein levels [15]. Despite many reports of Cks1 roles in transcription and the transcriptional regulation of Cks1 by other proteins, many gaps remain in our knowledge regarding the mechanisms of both these phenomena and require further investigation.

### 3.3. Cks1 Roles in Growth Signaling Pathways

The roles of Cks1 in growth factor signaling pathways have started to emerge with few studies suggesting the involvement of Cks1 in MAPK, JAK-STAT and NF-B pathways [13–24]. Despite its crucial role in cell cycle and cancer progression, mechanistic studies regarding Cks1 role in growth signaling mechanisms are lacking. Cks1 siRNA knockdown decreases ERK1/2 phosphorylation and triggers apoptosis in breast cancer cells MDA-MB-231 whereas its overexpression inhibits apoptosis and increases ERK1/2 phosphorylation hinting at a Cks1 modulated signaling event in this important pathway [25]. Another study showed that Cks1 shRNA mediated knockdown leads to decrease in ERK1/2, MEK and STAT phosphorylation in multiple myeloma cell lines KMS28PE, OCI-MY5 and XG-1 whereas forced overexpression of Cks1 activates ERK1/2 and STAT phosphorylation in the later two cell lines [21]. Surprisingly Skp2 knockdown or p27^Kip1^ overexpression caused suppression of ERK/MEK and JAK/STAT signaling indicating that Cks1 may be involved at a crucial node of regulation of signaling events independently of Skp2 and p27^Kip1^ [21].

Although the role of Cks1 upstream of these kinases at the level of Ras GTPases and Raf kinases has not been investigated, squamous cell carcinomas and lung adenomas derived from RasH2 mice have been shown to exhibit fluctuations in levels of Cks1 when treated with genotoxic agents like 7,12-dimethylbenz[a]anthracene (DMBA), urethane and N-ethyl-N-nitrosourea (ENU) [91]. It has also been shown that adaptor protein FGF receptor 2 (FRS2) associates with Cks1 and FGF dependent phosphorylation of FRS2 releases Cks1 and causes concomitant p27^Kip1^ degradation in 3T3 cells [24]. Recently the role of Cks1 in NF-B induced hepatocellular cancer has also been reported whereby Cks1 transcriptionally regulates IB and hence drives NF-B mediated IL-8 driven hepatocellular carcinoma [19]. Given that Cks1 is overexpressed in several different tumor types, its role as a signaling modulator remains a fruitful and unexplored avenue of research.

### 3.4. Other Roles of Cks1

Although the majority of research on Cks1 has been conducted using yeast and mammalian systems, there are a number of studies that have utilized other systems including *Xenopus laevis* [45], *Caenorhabditis elegans* [92], *Drosophila melanogaster* [93], *Branchiostoma belcheri tsingtauense* [94], *Leishmania Mexicana* [95] and *Patella vulgate* [96]. Cks1At, an *A. thaliana* homolog of Cks1 was shown by Montagu *et al.* to bind to arabidopsis CDKs Cdc2aAt and Cdc2bAt [97,98]. Further characterization of Cks1At showed that overexpression of Cks1 in *A. thaliana* lead to a reduction in leaf growth and root growth rates due to elongated G1 and G2 phases of the cell cycle [99]. Similarly some parasitic animal models have also been used in certain studies to isolate and characterize homologs of the Cks1 protein. For instance *Leishmania mexicana* has been used to isolate p12^Cks1^ which is the functional homolog of p13^Suc1^ counterpart found in yeast [95,100]. Not surprisingly this protein was found to interact with yeast and bovine cdc2 as well as with CRK1 and SBCRK1 which are cdc2-related kinases found in these parasites [95,100]. Recent studies with *Branchiostoma belcheri* (Amphioxus) have also suggested a potential developmental role of Cks1 [94]. Studies on Cks30 (Cks2 homologue in *Drosophila*) and Cks85A (Cks1 homologue in *Drosophhila*) have also demonstrated their role in female meiosis and mitosis during embryonic development, and maintenance of cell viability respectively [93,101–103].

In yet another report it was shown that Cks1 transcript levels gradually increase with increasing follicle size in bovine embryos possibly to ensure proper G2/M timing of cell cycle in the embryonic cells [82]. Skp2 and Cks1 also have been shown to play a crucial role in S/G2 phase transition during adipocyte differentiation in 3T3-L1 preadipocytes [104]. Yu *et al.* have also recently demonstrated that a balance between Cks1 and Cks2 regulates p27^kip1^ abundance and neuronal development in mouse embryonic cells [26]. Collectively these reports suggest novel roles of Cks family of protein in developmental biology which await further characterization. More recently Cks1 has also been shown to play a role in mitochondrial DNA replication regulating the mitochondrial single-stranded DNA-binding protein (mtSSB) function [105]. In another report demonstrating a p27^kip1^ independent Cks1 role it has been shown that Cks1 and Cks2 overexpression are involved in overcoming the DNA damage response following oncoprotein activation in breast cancer cells [27].

## 4. Cks1—Implications in Breast and Other Cancers

The importance of Cks1 in cancer progression can be understood given its pleiotropic roles in diverse biological processes that are known to be deregulated in cancer. Cks1 is overexpressed in a majority of human cancers and its expression is strongly correlated to tumor aggressiveness and dissemination of disease. Cks1 and its implications in cancers have been addressed herein by reviewing studies of Cks1 transcript levels, protein expression and gene amplification (the Cks1 gene is located on 1q21) in normal and/or cancer derived samples from patient cohorts of different cancer subtypes and their correlation to cancer clinicopathologic parameters. Because of its known role in the SCF-Skp2 complex many of these studies have also examined correlation of Cks1 expression to that of Skp2 and p27^Kip1^ or other cancer related markers (such as p53 and Ki-67) [106]. In general Cks1 expression is strongly correlated with Skp2 expression and inversely related to p27^Kip1^ expression [106–109]. However many studies including ours have reported different trends in expression pattern of these three genes emphasizing potentially underappreciated p27^Kip1^ independent mechanisms of Cks1 in cancer progression [110–112]. Another area of focus involves studies that attempt to determine the effect of Cks1 perturbation on cancer phenotypes (e.g. colony formation, migration and invasion, resistance to therapy etc.). For this discussion we broadly focus on two areas a) Cks1 expression, roles and implications in breast cancer, and b) Cks1 in other cancers.

### 4.1. Cks1 and Breast Cancer

We have found that normal mouse or rat tissues exhibit nearly undetectable levels of Cks1 protein, whereas both Cks1 mRNA and protein levels are very high in corresponding tumor tissues derived from mammary tumors excised from different murine models of mammary tumorigenseis (erbB2, c-myc and polyoma middle-T (PyMT) driven transgenic mice) and in carcinogen-initiated rat models [110]. In agreement with these studies a previous analysis of global gene expression patterns of mammary tumors initiated by the PyMT oncogene expressed in the context of five different genomic backgrounds revealed that Cks1 expression was greatly increased in PyMT-transgenic mammary tumors [113]. Interestingly, we found that p27^Kip1^ levels were not reduced, and were in fact slightly higher in mammary tumors initiated by erbB2, PyMT and MNU [110]. It is also known that the relative abundance of Cks1 SAGE tags in breast carcinoma tumor samples is higher compared to that in normal human mammary epithelium [114].

In one of the first studies that examined the role of Cks1 overexpression in human breast cancer Slotky *et al.* reported that Cks1 overexpression is associated with loss of tumor differentiation, younger age of patients, lack of expression of estrogen and progesterone receptors, decreased disease-free and overall survival [109]. Furthermore Cks1 and Skp2 expression was increased by estradiol in estrogen-dependent cell lines but were downregulated by tamoxifen [115]. In fact, in agreement with these findings we have previously demonstrated that stable overexpression of Cks1 in human breast carcinoma MCF-7 cells confers resistance to Faslodex (ICI-182780) whereas Cks1 knockdown led to a decrease in colony formation in estrogen-containing medium [110].

We have also shown that Cks1 depletion in MCF-7 breast cancer cells blocks cell cycle progression induced by both estrogen dependent and growth factor dependent pathways [70]. Cks1 depletion not only slows progression through G1-S, but also blocked their entry into M phase. Cks1 silencing also leads to a rapid loss of Skp2, concomitant increases in p130/Rb2 and p27^Kip1^, and marked reduction in the level of CDK1, which is essential for M phase entry. Interestingly Cks1 loss does not alter cell cycle profiles in human mammary epithelial cells or normal human lung fibroblast suggesting that targeting it might provide selectivity [70].

Given its importance in cancer progression it is not surprising that significant efforts have been directed to target Cks1 as a potential anti cancer target. Fluoxetine and Vorinostat are two drug candidates that have been shown to induce an accumulation of p27^Kip1^ and p21^Cip1^ and consequently cause cell cycle arrest through a Cks1 dependent mechanism in MDA-MB-231 breast cancer cells [84,116]. A DNA-microarray analysis to evaluate the anticancer effects of a dietary supplement MycoPhyto^®^ Complex (MC) has revealed that MC inhibits expression of Cks1 in MDA-MB-231 breast cancer cells suggesting a potential role for Cks1 targeting by chemopreventives [117]. An *in silico* screen targeting the phospho-p27^Kip1^ binding pocket led to development of a family four specific small molecule inhibitors collectively referred to as SKPins (C1, C2, C16, and C20) that prevent the ubiquitination and degradation of p27^Kip1^ and p21^Cip1^ exclusively by perturbing critical interactions that allow phospho-p27^Kip1^ to bind in the pocket formed by Skp2-Cks1 [118–120]. This ultimately ensues in a cell type specific block in G1 or G2/M phase in T47D and MCF-7 respectively. Not surprisingly mutations in key residues mediating these contacts (for instance Cks1 Q52L) can reverse the inhibitory activity of some these compounds [120].

### 4.2. Cks1 in Other Malignancies

Expression analyses utilizing microarray platforms, qPCR studies and IHC analyses have revealed that Cks1 is overexpressed in different subtypes of lymphoma including mantle cell lymphoma (MCL) and mantle cell lymphoma blastoid variant (MCL-BV), and contributes to development of disease and resistance to cancer chemotherapy [121–124]. Studies delineating the precise mechanisms of Cks1 role in development and progression of lymphomas are still lacking however an important study in this regard has shown that Myc induced Cks1 can drive development of disease [81]. Loss of Cks1 markedly delays lymphoma development and dissemination of disease in the Eμ-Myc transgenic mouse lymphoma model [81]. Furthermore inhibition of PI3K/Akt pathways can decrease MCL growth by inducing p27^Kip1^ accumulation through Cks1 and Skp2 downregulation [17]. In yet another report it was found that retinoic acid downregulates the expression of the Cks1 and Skp2 proteins thus slowing down p27^Kip1^ degradation in lymphoblastoid B cell lines [125].

Expression studies have employed fluorescent *in situ* hybridization and other methods to ascertain prevalence and prognostic significance of Cks1 gain following 1q21 amplification in multiple myeloma (MM) progression [126–129]. Cks1 gain is associated with transformation from benign monoclonal gammopathy of undetermined significance (MGUS) to more aggressive forms MM and plasma cell leukemias (PCL) and a shorter disease free survival [127]. Like lymphoma, little is known about detailed mechanisms of Cks1 in MM cells. However a study utilizing microarray based gene expression analysis of CD138 enriched plasma cells from MM patients undergoing melphalan based high dose therapy has suggested Skp2 and p27^Kip1^-dependent and independent mechanisms that fuel into multiple myeloma progression [130]. In fact Cks1 overexpression leads to multidrug resistance in multiple myeloma and stimulates STAT3 and MEK/ERK signaling pathways [21].

Cks1 is also highly overexpressed in the majority of different cancer subtypes afflicting the gastrointestinal system and in most cases is strongly correlated to increased Skp2 expression and reduced p27^Kip1^ expression. Cks1 has been implicated in development of oral squamous cell carcinoma [131–133], salivary gland tumors [106], esophageal carcinomas [80,134,135], gastric carcinoma [20,136], colorectal carcinoma [108], gall bladder carcinoma [137] and hepatocellular carcinoma [19, 138–142]. Similarly Cks1 is believed to play a role in development and progression of several other types of cancers such as endometrial cancer [143], ovarian tumors [144–147], prostate cancer [148], testicular cancer [149], non small cell lung carcinomas (NSCLC) [111,150], cutaneous squamous cell carcinoma [151], melanoma [15], urothelial carcinoma and renal cell carcinomas [152,153], glioblastoma and CNS tumors [154,155], head and neck carcinoma [156], fibrosarcoma [157], and myxofibrosarcoma [158]. In many of these studies there is often a distinct correlation between Cks1 expression and clinicopathologic features such as tumor grade, stage, metastasis, loss of tumor differentiation patient prognosis and cancer free survival.

## 5. Conclusion

In conclusion, cellular and biochemical studies providing a clearer understanding of Cks1 and its function in cancer biology is likely to yield attractive avenues for therapeutic intervention.

## Figures and Tables

**Table 1:** Cks1 structural features and their functional implications.

Property	Residue	Region and Contacts
Anion Binding Pocket (Binds pThr187 of p27^Kip1^)	K11	*β*1
	R20	*β*2
	S51	Between *α*2 and *β*3
	W54	*β*3
	R71	*β*4 (all involved in charge-stabilized H-bonding)

Cdk binding region	Y12	*β*1 (hydrophobic)
	Y19, H21, M23	*β*2 (hydrophobic)
	Y57, M58	Between *β*2 and *β*3
	H60, P62, I66, L68, R70	*β*4 (hydrophobic)
	E63	*β*4 (H-bonding)

Skp2 binding region	E40, S41, E42, N45	*α*2 (H-bonding)
	L31, P33	*α*1 (van der Waals)
	H36, M38	*α*2 (van der Waals)

Dimer interaction sites	Y8	*β*1 (H-bonding)
	I6, Y7, D10	*β*1
	H21	*β*2
	M23, Q49	between *α*2 and *β*3
